# Supplementary material for: Towards unified AI-driven fracture mechanics: the extended deep energy method (XDEM)
Source: Nat Commun. 2026 Aug 15;17:8492. doi: 10.1038/s41467-026-76748-1 (PMC13484471; doi:10.1038/s41467-026-76748-1)
Supplement: Supplementary file 3 [file 41467_2026_76748_MOESM3_ESM.zip › pic/discussion/loss_landscape/a05_loss_comparison.pdf]

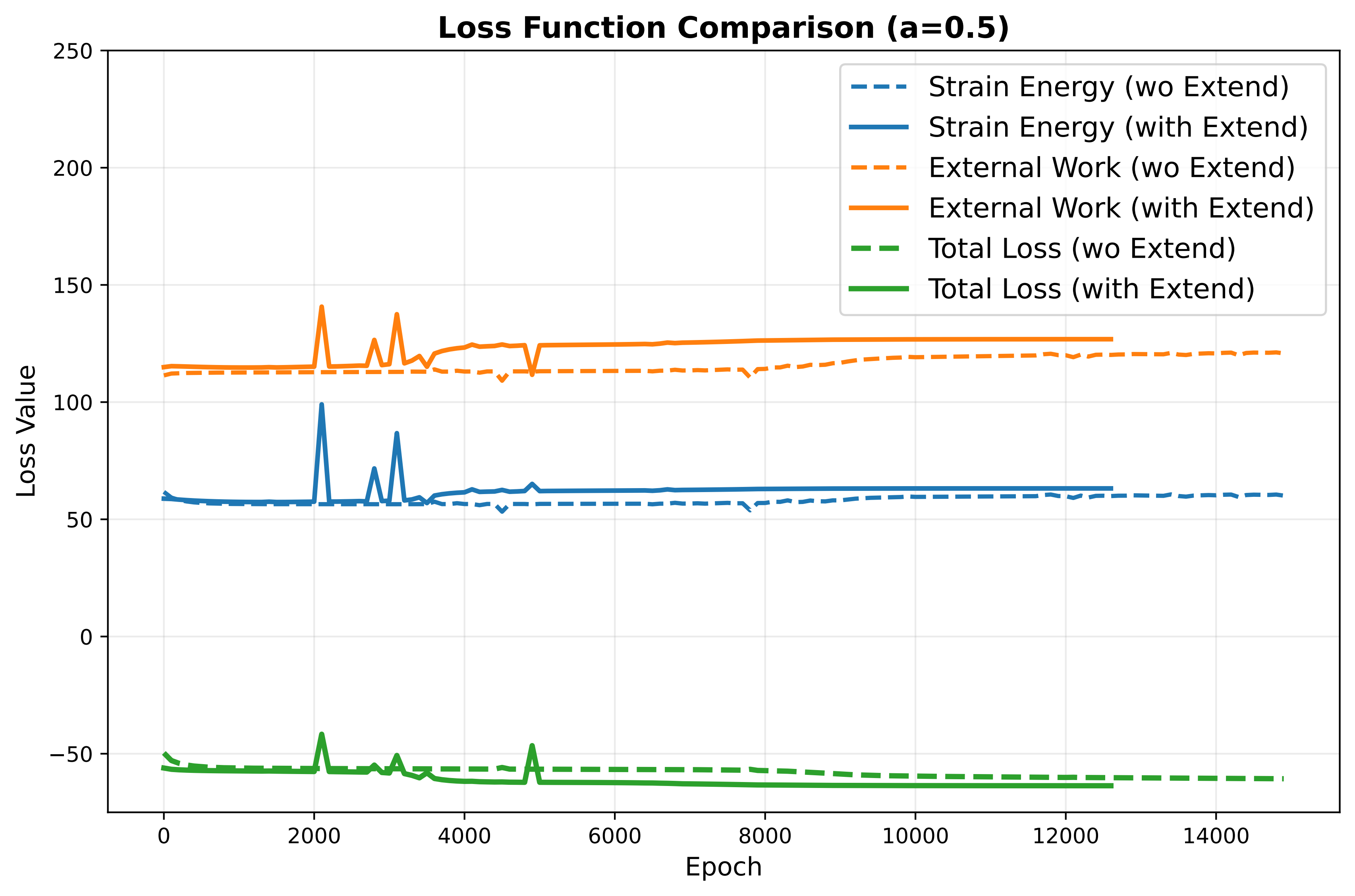

Loss Function Comparison (a=0.5)
Strain Energy (wo Extend)
Strain Energy (with Extend)
External Work (wo Extend)
External Work (with Extend)
Total Loss (wo Extend)
Total Loss (with Extend)
Loss Value
Epoch
250
200
150
100
50
0
−50
0
2000
4000
6000
8000
10000
12000
14000
